# Partial STX11 deficiency due to a hypomorphic variant—self-limiting inflammatory disease preceding HLH onset

**DOI:** 10.70962/jhi.20250100

**Published:** 2025-10-07

**Authors:** Tahereh Noori, Anit Kaur, Altaf Hussain Kambay, Aaqib Zaffar Banday, Ilia Voskoboinik

**Affiliations:** 1 Killer Cell Biology Laboratory, Peter MacCallum Cancer Centre, Melbourne, Australia; 2 Department of Immunopathology, Post Graduate Institute of Medical Education and Research, Chandigarh, India; 3 Department of Paediatrics, Government Medical College Srinagar, Srinagar, India; 4 Cancer Cell Death Laboratory, Cancer Immunology Program, Peter MacCallum Cancer Centre, Melbourne, Australia; 5 Sir Peter MacCallum Department of Oncology, University of Melbourne, Parkville, Australia

## Abstract

A hypomorphic STX11 variant (L135P) was identified in a patient with suspected atypical familial hemophagocytic lymphohistiocytosis (HLH). Functional validation confirms partial cytotoxic deficiency associated with a self-limiting inflammatory phenotype that may precede the clinical onset of full-blown HLH.

## Teaser

Recently developed *in vitro* technology was used to define the function of a previously uncharacterized STX11 mutation L135P present in a patient with atypical FHL. The results confirmed the FHL diagnosis and enabled clinicians to proceed with assurance to hematopoietic stem cell transplantation.

## Introduction

Familial hemophagocytic lymphohistiocytosis (FHL) is a potentially fatal autosomal recessive immune dysregulation disorder caused by dysfunction of cytotoxic lymphocytes. To date, biallelic pathogenic mutations in four genes—*PRF1* (perforin), *UN13D* (MUNC13-4), *STX11* (syntaxin-11), and *STXBP2* (MUNC18-2)—have been associated with FHL 2, 3, 4, and 5, respectively (1, 2). The result is failure to release functional perforin, an indispensable component of the cytotoxic machinery of T lymphocytes (CTL) and natural killer (NK) cells. Mutations in *PRF1* lead to release of inactive perforin, while mutations in the other genes lead to failed secretion of wild-type (WT) perforin.

Mutations in the target soluble N-ethylmaleimide-sensitive factor attachment protein receptor (t-SNARE) protein STX11 are responsible for about 15% of FHL cases (1). Through its interaction with STXBP2, STX11 facilitates the exocytic release of cytotoxic granule contents. Until recently, the presence of biallelic mutations in one of the four FHL-associated genes, along with hemophagocytic lymphohistiocytosis (HLH) symptoms, was considered sufficient for an FHL diagnosis and to initiate allogenic bone marrow transplantation as the only curative option. However, simply assuming that a variant of uncertain significance (VUS) is pathogenic without testing its effect on function may unnecessarily expose a patient to the potentially life-threatening risks of transplantation (3).

In this clinical study, we used recently developed *in vitro* technology to define the function of a previously uncharacterized *STX11* mutation present in a patient with suspected atypical FHL. This information proved critical for confirming the FHL diagnosis and enabled clinicians to proceed with assurance to hematopoietic stem cell transplantation.

## Results

**Figure 1.**
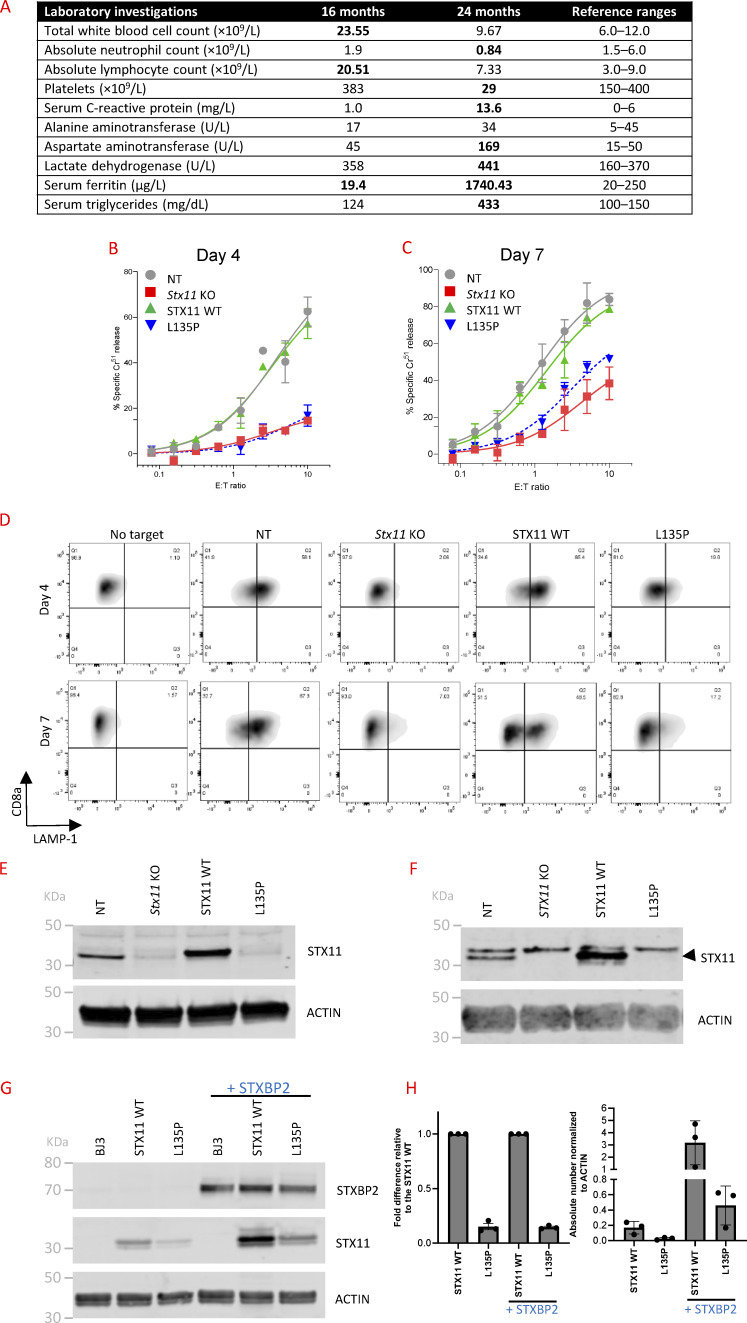
**The effect of the L135P mutation on STX11 function. (A)** Clinical data from the patient at first presentation (16 mo old) and second presentation (24 mo old). **(B–C)** A ^51^Cr-release assay performed on days 4 (B) and 7 (C) using activated *Stx11*-KO murine CD8^+^ T cells show a rescue of cytotoxic function when WT STX11 is expressed, but reduced cytotoxicity when STX11-L135P is expressed. The assay was done using CD8^+^ T cells from Bl/6.OT1 mice following their incubation with SIINFEKL-treated syngeneic EL4 target cells. Data represent mean ± SEM from *n* = 3 independent experiments. NT, non-targeting gRNA-transfected cells (as per [3]). **(D)** Degranulation was measured by the externalization of CD107a on CD8^+^ T cells from BL/6.OT1 mice following their incubation with SIINFEKL-treated syngeneic EL4 targets. Reduced levels of degranulation were observed in *Stx11*-KO CD8^+^ T cells expressing STX11-L135P on both days 4 and 7, compared to cells expressing WT STX11 (STX11 WT). Shown is a representative result of three independent experiments. NT, non-targeting gRNA-transfected cells. **(E–H)** Western immunoblot analysis of recombinant human STX11 in various cell types. β-actin was used as a loading control. NT, non-targeting gRNA-transfected cells. **(E)** Expression of recombinant STX11-WT and STX11-L135P in *Stx11*-KOmurine primary CD8^+^ T cells. Shown is a representative result of three independent experiments. NT, non-targeting gRNA-transfected cells. **(F)** Expression of recombinant STX11-WT and STX11-L135P in *STX11*-KO human primary CD8^+^ T cells. Shown is a representative result of two independent experiments. NT, non-targeting gRNA-transfected cells. **(G)** Expression of recombinant STX11-WT and STX11-L135P in human fibroblast cell line BJ3 with or without transduction of recombinant STXBP2-WT. Shown is a representative result of three independent experiments. **(H)** Quantification of three independent western blots, one of which is shown in Fig. 1 G. The left plot shows the fold increase in expression of the STX11-L135P mutant relative to STX11-WT in BJ3 cells, either without STXBP2 (7.0 ± 1.1, mean ± SEM, *N* = 3) or with STXBP2 overexpression (6.9 ± 0.4, mean ± SEM, *N* = 3). The right plot shows actin-normalized expression levels of STX11-WT and STX11-L135P, with expression differences of 17.9 ± 1.5 (without STXBP2 overexpression) and 17.7 ± 1.4 (with STXBP2 overexpression) (mean ± SEM, *N* = 3), respectively. E:T, Effector:target. Source data are available for this figure: SourceData F1.

A 16-mo-old boy, born to consanguineous parents (first cousins), presented with intermittent fever, hepatosplenomegaly, cervical lymphadenopathy, and failure to thrive (8.3 kg, −2.16 z). Investigations showed mild nutritional anemia, lymphocytosis (CD3^+^ T cell and CD8^+^ cytotoxic T cell lymphocytosis), decreased serum ferritin, and decreased B cell counts. A detailed workup for infections was noncontributory. Peripheral blood double-negative T cells were mildly elevated (2.93% of T lymphocytes), and bone marrow aspiration/biopsy showed mild hemophagocytosis. However, the diagnostic criteria of FHL or autoimmune lymphoproliferative syndrome remained unfulfilled at the initial presentation (Fig. 1 A). Although no specific therapy could be utilized (except hematinics), he improved spontaneously (fever subsided, hepatosplenomegaly/lymphadenopathy improved) and did not require any hospital visits for the next 8 mo. Whole-exome sequencing performed at first presentation showed a homozygous missense variant in the *STX11* gene (c.404T>C, p.Leu135Pro). Unaffected parents and a younger sibling were heterozygous carriers of the variant. At 8 mo of follow-up, the child presented again with a 1-wk history of fever, worsening hepatosplenomegaly, and generalized lymphadenopathy. Investigations showed neutropenia, thrombocytopenia, hyperferritinemia, hypertriglyceridemia, and elevated circulating levels of aspartate aminotransferase and lactate dehydrogenase, resulting in fulfillment of the familial HLH criteria. He was treated with intravenous pulse steroids, cyclosporine, and etoposide. He has been in remission for 13 mo. NK cell cytotoxicity or degranulation could not be tested due to the lack of an appropriate hospital setup.

Given that the *STX11* variant c.404T>C, p.Leu135Pro was initially classified as a VUS (see Discussion), we assessed its function using our previously established protocol (3). On day 4 of cell activation, the mutation showed no effect on the cytotoxic function of *Stx11-KO* CTLs (Fig. 1 B), but the degranulation was higher than in *Stx11*-deficient cells (Fig. 1 D). Considering this and the fact that the patient had only developed transient symptoms at 16 mo of age, we extended the analysis to day 7 of cell activation. At this later time point, a small but measurable level of cytotoxicity compared to the *Stx11-KO* cells was observed (Fig. 1, C and D) suggesting that the mutant protein retained some residual function. This finding was consistent with mutations associated with an atypical FHL (3).

Despite this partial functional activity, STX11-L135P protein expression was nearly undetectable in mouse (Fig. 1 E) or human CD8^+^ T cells lacking endogenous *STX11* (Fig. 1 F). Since the stability of STX11 is regulated by STXBP2 and the L135P is located within the STXBP2 interaction domain, it was possible that the mutation impairs the protein–protein interaction, resulting in the degradation of STX11-L135P. Alternatively, L135P could intrinsically destabilize the protein structure. To explore these possibilities, we expressed the STX11-L135P mutant in *Stxbp2*/*Stx11* double-KO OT-1 CTLs and *STXBP2/STX11* double-KO hCD8^+^ T cells to determine whether the absence of Stxbp2 and STXBP2, respectively, would alter the expression of STX11-L135P. If expression remained unchanged in the absence of Stxbp2/STXBP2, it would suggest the mutation primarily affects protein intrinsic stability. However, we still detected no measurable levels of the STX11-L135P protein (data not shown).

We then took a different approach and overexpressed the L135P mutant and WT STX11 in the human dermal fibroblast cell line, BJ3, which expresses minimal endogenous STX11 and STXBP2 (Fig. 1 G). In this context, STX11-L135P was clearly detectable but expressed at a lower level than STX11-WT. We reasoned that if the instability of the mutant resulted from its failed interaction with STXBP2, then overexpressing STXBP2 in STX11-transduced cells would stabilize the WT protein but not the mutant. However, when we overexpressed STXBP2, the expression of both WT and mutant STX11 increased proportionately (Fig. 1, G and H), suggesting that the L135P mutant was intrinsically unstable but could still interact with STXBP2.

Taken together, these findings suggest that the intrinsic instability of L135P plays a key role in the significantly reduced expression of the mutant compared to the WT protein. However, its retained ability to interact with STXBP2 is responsible for the partial activity of cytotoxic lymphocytes expressing the mutant protein.

## Discussion

Using our recently published experimental protocol, we assessed the pathogenicity of a *STX11* missense mutation L135P (3). Reconstitution of the mutant protein in *Stx11*-KO CD8^+^ T cells resulted in severely reduced, but not ablated, cytotoxicity and degranulation compared to STX11-WT, explaining the patient’s late onset, atypical presentation, and transient symptoms (Fig. 1, B–D). In the absence of functional assays on patient’s NK cells, these functional data were critical for confirming an FHL diagnosis and guiding appropriate treatment.

Distinguishing between familial and acquired forms of HLH is essential for clinical decision making. Unnecessary transplantation in patients with acquired HLH has been associated with increased mortality compared to transplanted patients with confirmed FHL. However, only a limited number of mutations in FHL-associated genes have been functionally characterized, and diagnoses are often based on the presence of genetic variants of uncertain significance. Several factors may limit the reliable analysis of patient samples in suspected cases of FHL, such as the availability of fresh blood samples, acute active disease (the blood is typically analyzed in severely ill patients), certain treatments (e.g., cyclosporine), and the lack of appropriate control samples, particularly in infants. The recently developed *in vitro* assay for testing FHL-associated mutations (3) addresses this diagnostic gap by providing enough cells, generated under standardized conditions, for precise functional testing and assessment of mutant protein stability. Indeed, we were able to confirm an atypical FHL diagnosis despite the absence of conventional NK cell assays and a patient blood sample, neither of which was feasible due to the hospital’s remote location and lack of appropriate clinical laboratory facilities.

Our study highlights the occurrence of a self-limiting inflammatory phenotype in partial STX11 deficiency caused by L135P variant, preceding the onset of HLH. A recent paper from India (4) describes a patient with a homozygous STX11-L135P variant who exhibited a similar inflammatory illness (fever and edema) at 2 years of age that resolved spontaneously, prior to development of HLH at 4 years of age. Although the number of reported patients with STX11-L135P remains limited, our study contributes to emerging genotype–phenotype correlations in STX11 deficiency. Specifically, the hypomorphic L135P variant appears to be associated with a milder, self-limiting inflammatory illness prior to the onset of full-blown HLH (5).

The L135P variant was first identified in our patient during an initial presentation in mid-2023 with an inflammatory illness, at which time the diagnostic criteria for FHL were not fulfilled. It was initially classified as a VUS according to the American College of Medical Genetics and Genomics and the Association for Clinical Genomic Science guidelines, based on the following criteria: PM2_moderate: absent in population databases, PM3_supporting: noted in one affected patient previously (5), and PP3_supporting: deleterious by in silico tools. With our report and evidence available thus far, the variant is currently (mid-2025) classified as likely pathogenic—PS3_moderate: functional evidence of pathogenicity, PM2_moderate: absent in population databases (gnomAD v.4.1.0, last accessed March 18, 2025), PM3_moderate: noted in two affected unrelated patients previously (4, 5), PP3_supporting: deleterious by in silico tools, and PP4_supporting: consistent phenotype.

As all three patients with the STX11-L135P variant reported to date (including our case) are from India, a founder effect for this variant appears likely. The functional validation of the damaging impact of L135P on STX11 function (as demonstrated in our study) thus carries significant clinical implications: the discovery of residual cytotoxic activity suggested that the patient can be stabilized with pharmacological intervention as required, allowing for potential delay of transplantation. Additionally, our study highlights the feasibility of a novel *in vitro* system for testing VUSs in genes associated with FHL. Such functional characterization has substantial therapeutic and prognostic relevance, including the potential for future prenatal testing, particularly in resource-limited settings where flow cytometry and other advanced techniques may be unavailable, as was the case in our study.

## Materials and methods

For full details of the methodologies and reagents used in this paper, please refer to (3). Briefly, endogenous *Stx11* was disrupted in purified naive murine BL/6.OT-1 CD8^+^ T cells using CRISPR/Cas9 delivered by nucleofection. These cells were transduced with a retrovirus to express recombinant human WT and L135P-STX11, along with a GFP reporter. The OT-1 CD8^+^ T cells were activated with the cognate ova peptide (SIINFEKL), and transduced cells were sorted based on equivalent GFP fluorescence. BJ3 cells were also transduced with retrovirus vectors to express recombinant human WT or L135P-STX11 protein or STXBP2 along with a BFP reporter. All cells were sorted based on identical GFP and BFP fluorescence levels. The cytotoxic activity of the CD8^+^ T cells was assessed using a 4-h ^51^Cr-release assay, and their degranulation capacity was measured using LAMP-1/CD107a externalization assay. Western immunoblotting was performed as described in (3). All relevant experiments were approved by the Peter MacCallum Cancer Centre’s Animal and Human Experimentation Ethics Committees.

## Supplementary Material

SourceData F1 is the source file for Fig. 1.

## Data Availability

The data are available from the corresponding author upon reasonable request.
