# Supplementary figures and images for: Partial STX11 deficiency due to a hypomorphic variant—self-limiting inflammatory disease preceding HLH onset
Source: J Hum Immun. 2025 Oct 7;1(4):e20250100. doi: 10.70962/jhi.20250100 (PMC13177436; doi:10.70962/jhi.20250100)

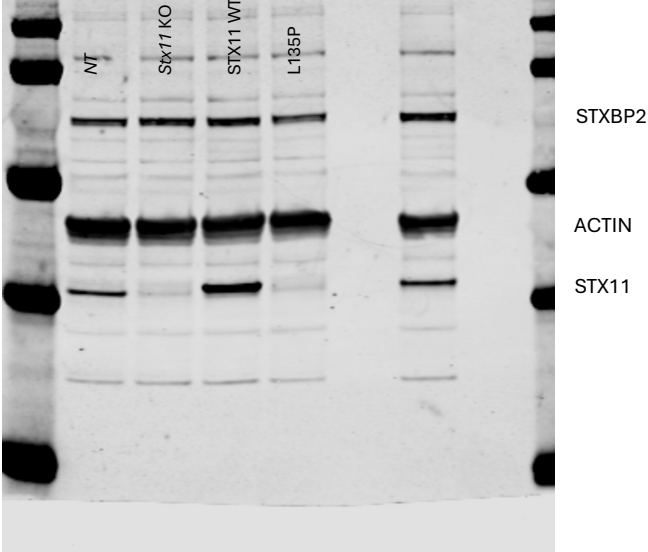

Source Figure 1E

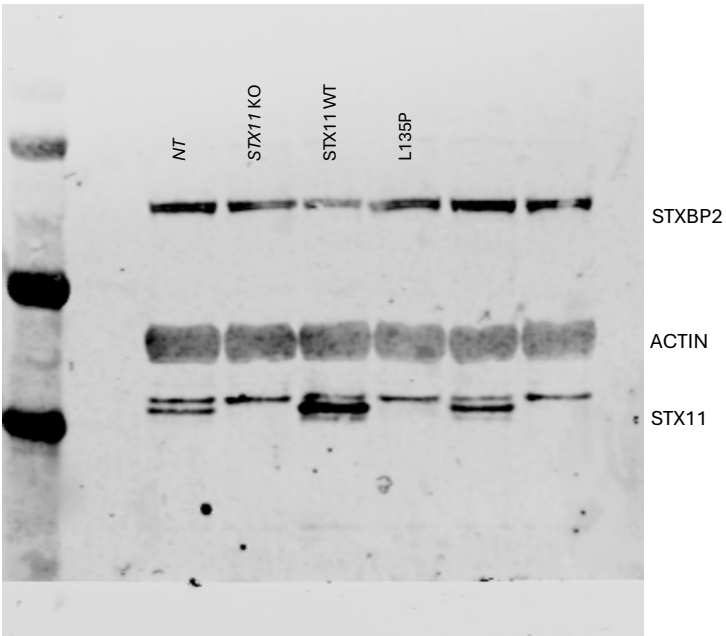

Source Figure 1F

#1

#2

#3

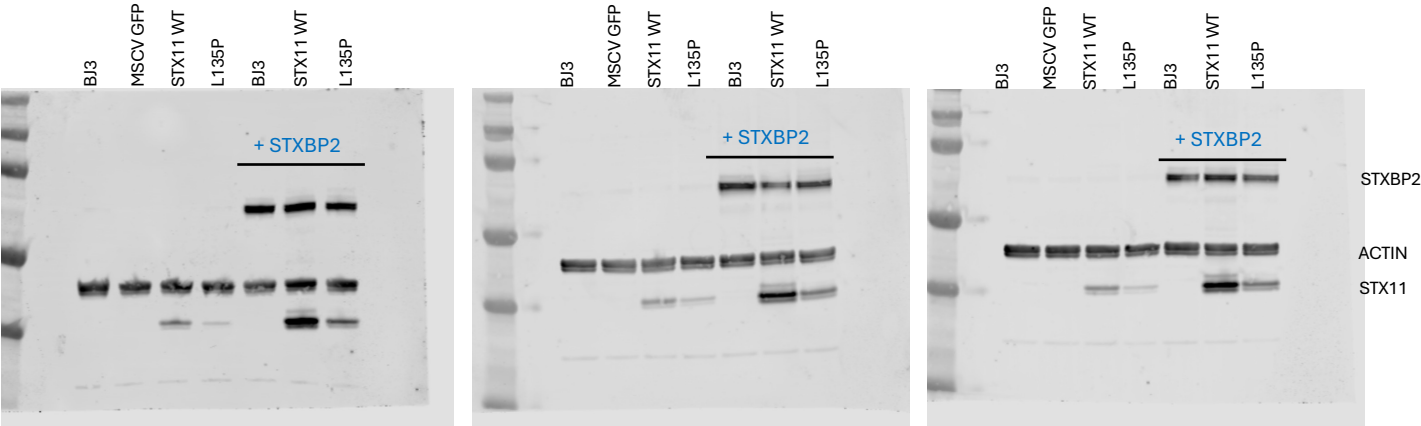

Source Figure 1G

Supplement: SourceData F1 — is the source file for Fig. 1. [file jhi_20250100_sourcedataf1.pdf]
